# Spatial Variations in Dengue Transmission in Schools in Thailand

**DOI:** 10.1371/journal.pone.0161895

**Published:** 2016-09-26

**Authors:** Pitcha Ratanawong, Pattamaporn Kittayapong, Phanthip Olanratmanee, Annelies Wilder-Smith, Peter Byass, Yesim Tozan, Peter Dambach, Carlos Alberto Montenegro Quiñonez, Valérie R. Louis

**Affiliations:** 1 Institute of Public Health, Heidelberg University Medical School, Heidelberg, Germany; 2 Center of Excellence for Vectors and Vector-Borne Diseases, Faculty of Science, Mahidol University at Salaya, Nakhon Pathom, Thailand; 3 Faculty of Science and Technology, Rajabhat Rajanagarindra University, Chachoengsao, Thailand; 4 Epidemiology and Global Health, Department of Public Health and Clinical Medicine, Umeå University, Umeå 90187, Sweden; 5 Lee Kong Chian School of Medicine, Nanyang Technological University, Singapore, Singapore; 6 Medical Research Council/Wits University Rural Public Health and Health Transitions Research Unit (Agincourt), School of Public Health, Faculty of Health Sciences, University of the Witwatersrand, Johannesburg 2193, South Africa; 7 College of Global Public Health, New York University, New York, New York, United States of America; International Atomic Energy Agency, AUSTRIA

## Abstract

**Background:**

Dengue is an important neglected tropical disease, with more than half of the world’s population living in dengue endemic areas. Good understanding of dengue transmission sites is a critical factor to implement effective vector control measures.

**Methods:**

A cohort of 1,811 students from 10 schools in rural, semi-rural and semi-urban Thailand participated in this study. Seroconversion data and location of participants’ residences and schools were recorded to determine spatial patterns of dengue infections. Blood samples were taken to confirm dengue infections in participants at the beginning and the end of school term. Entomological factors included a survey of adult mosquito density using a portable vacuum aspirator during the school term and a follow up survey of breeding sites of *Aedes* vectors in schools after the school term. Clustering analyses were performed to detect spatial aggregation of dengue infections among participants.

**Results:**

A total of 57 dengue seroconversions were detected among the 1,655 participants who provided paired blood samples. Of the 57 confirmed dengue infections, 23 (40.0%) occurred in students from 6 (6.8%) of the 88 classrooms in 10 schools. Dengue infections did not show significant clustering by residential location in the study area. During the school term, a total of 66 *Aedes aegypti* mosquitoes were identified from the 278 mosquitoes caught in 50 classrooms of the 10 schools. In a follow-up survey of breeding sites, 484 out of 2,399 water containers surveyed (20.2%) were identified as active mosquito breeding sites.

**Discussion and Conclusion:**

Our findings suggest that dengue infections were clustered among schools and among classrooms within schools. The schools studied were found to contain a large number of different types of breeding sites. *Aedes* vector densities in schools were correlated with dengue infections and breeding sites in those schools. Given that only a small proportion of breeding sites in the schools were subjected to vector control measures (11%), this study emphasizes the urgent need to implement vector control strategies at schools, while maintaining efforts at the household level.

## Introduction

The global incidence of symptomatic and asymptomatic dengue infections per year is estimated at about 400 million, and it is expected to rise [1]. More than half of the world’s population lives in dengue endemic areas with more than 70% of those at risk reside in Asia Pacific region, and further geographic expansion of dengue is anticipated because of globalisation, urbanisation, and climate change [2–5]. Thailand is experiencing steadily increasing urbanization; the urban population increased from roughly 45% of the total population in 2000 to 49% of total population in 2014 [6]. This transition from rural towards urban patterns of living has directly affected dengue infection rates [4]. In the absence of vaccines and antiviral therapies specifically targeting the dengue virus, dengue prevention and control efforts have relied heavily on vector control; and these efforts have mostly proved ineffective in the effort to control dengue transmission or outbreaks. Therefore, complementary vector control strategies are urgently needed to protect at-risk populations in endemic areas.

In Thailand, dengue is a serious public health concern and a leading cause of childhood hospitalisations [7]. The average age of dengue infected patients has progressively shifted towards older children and younger adults in the past two decades with the age group 10–14 years carrying the greatest burden [8]. Without adequate care, the infection-fatality rate for severe dengue can be as high as 20%, but this can be reduced to less than 1% with good patient management [7,9,10].

*Aedes aegypti* and *Aedes albopictus* are the predominant vectors for dengue, and both vectors are daytime biters [11–14]. Given this daytime biting behaviour, it is important to identify dengue transmission sites in order to better target vector control interventions and hence increase their effectiveness. Several studies have claimed that the majority of dengue infections occur peri-domestically [15–17], and vector control has therefore been targeted at residential areas. While vector control in schools has been proven effective in reducing dengue infections in Thailand, schools are still generally neglected as implementation sites [18,19]. Given that school-aged children spend a large part of their day at school, it also makes sense to equally target schools using dengue vector control measures, since controlling dengue infection in children is a priority. Although schools have previously been suggested as potential sites for dengue transmission based on adult vector and larvae surveys, results from many studies have been inconsistent [20–22]. A similar study in Thailand focusing on dengue hemorrhagic fever, using active disease surveillance and vector survey methods, suggested that dengue transmission was most likely to happen in households [23]. So far, there have been no robust data to validate dengue infection for investments in vector-based control strategies targeting schools [24].

The main objective of this study is to explore whether schools are likely dengue transmission sites for school children by assessing dengue infections and *Aedes* vector abundance in schools. Using a combination of seroconversion data, households’ geographic information data, and participants’ classroom data, we aim also to explore possible dengue clustering among school children at the classroom-level.

## Materials and Methods

In order to determine whether schools were active sites for dengue transmission, geospatial analysis of confirmed dengue infections and surveys of *Aedes* vectors were carried out in 10 schools. First, serological surveys were carried out in children from 10 selected schools to identify dengue infections. Second, we surveyed both adult and immature stages of *Aedes* vectors in the same selected schools to assess the potential risk of dengue transmission in the schools. Third, mapping and clustering analysis of confirmed dengue infections were performed at both school and household levels.

### Study setting and participants

**Fig 1:**
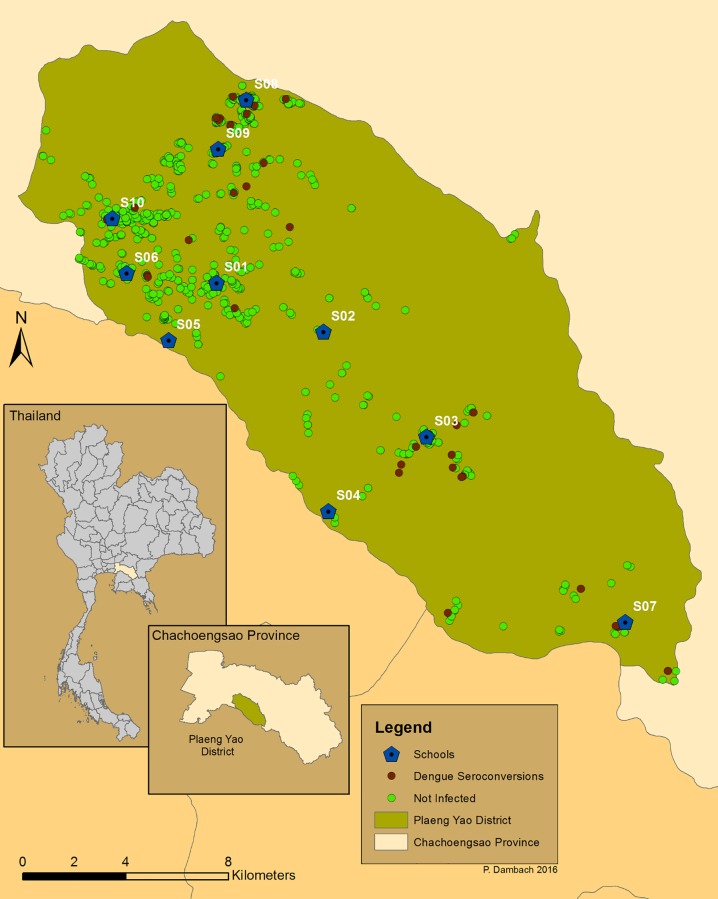
Map of the study area in Chachoengsao Province, indicating the location of schools and participants’ residents. Each point represents a school, or a household of each participating student, (either serologically positive and negative).

The study was conducted in Plaeng Yao District of Chacheongsao Province in eastern Thailand. Located in an area of high dengue endemicity, Plaeng Yao District had a population of 17,963 in 7,233 households in 2013, and covering an area of 236 km^2^ [25]. The district is divided into four sub-districts with a total of 48 villages. The economy of Plaeng Yao District was historically based on agriculture such as rice paddies, fruit plantations, and livestock. However, over the past decade, the district has become more industrialized with farms turning into factories and rural areas transitioning into semi-rural and semi-urban areas. This study was carried out in 10 out of 24 schools in the District as shown in Fig 1. All students in the 10 schools were eligible to participate in this study. However, transferred students, students without consent forms, and students without paired-blood samples for seroconversion test due to unavailability for either blood sample were excluded from the study. A total of 1,811 students were enrolled in the trial, however, only 1,655 pairs of blood samples were obtained at the beginning and at the end of the school term. Hence our analyses are based on 1,655 students (91.4% of those enrolled).

### Ethical Considerations

The protocol for this study was reviewed and approved by the Ethical Review Board of Mahidol University and Heidelberg University. Participant information, such as classroom number, grade, age, address, and phone number, was collected from school records. Parental written consent was obtained for all participating students. In the case of children aged 12 years and older, individual written assent was also obtained.

### Dengue infection monitoring

The rainy season is usually from July to October in this area of Thailand, and the school term runs from June to November. We collected blood samples via finger-prick (<0.2 ml) from participating students at the beginning and end of the school term (June and November 2012). Dengue IgG enzyme-linked immunosorbent assays (ELISA) were first performed for all paired samples. If one or both samples (from the beginning and the end of the school term) were positive, paired samples were further assessed by monoclonal antibody (MAb)-based capture ELISAs, in which Dengue IgG indirect ELISAs were performed using a purified 2H2 monoclonal antibody for coating plates on paired samples. Results were expressed in terms of positive to negative ratio as described by Johnson *et al*. [26].

### Entomological monitoring

At baseline in June 2012, indoor adult mosquito surveys were conducted by trained staff inside five randomly selected classrooms in each participating school, using locally made portable vacuum aspirators, for 15 minutes per room. For quality insurance purpose, the same operator conducted the surveys for all schools. Mosquitoes collected from classrooms were transferred to the Center of Excellence for Vectors and Vector-Borne Diseases (CVVD), Faculty of Science, Mahidol University to be counted and identified for species.

In order to identify and characterize the type of breeding sites of *Aedes* vectors at the schools, monthly larvae/pupae follow-up surveys were conducted from May 2013 to June 2014 by members of staff from Mahidol University together with public health volunteers trained to use and fill in the data collection form. For each school, the school grounds and neighbouring residential areas within 1 km were inspected. Surveys recorded the type and number of breeding containers with water, the presence of *Aedes* larvae and pupae, and the vector control measures in place. Only the sites that actually contained water were recorded as potential breeding sites.

### Data analysis

Entomological and serological data were handled using Microsoft Excel 2011. STATA 12 (Stata Corporation, College Station, TX, USA) was used for data analysis, including the **cij** command for confidence intervals of proportions based on the Jeffreys uninformative prior, because of the relatively small numbers and rates involved. Statistical significance was taken as p<0.05. Data that were not normally distributed, such as mosquito counts, were log-transformed. Regression of dengue infection data in relation to entomological parameters was weighted by the number of participating students in each school. The dengue infections were further disaggregated to classroom level within schools. Poisson goodness-of-fit statistics were used to assess the extent to which these dengue infections were clustered among classrooms. Although the numbers were too small to meaningfully compare dengue infection rates between classrooms, it was possible to consider, within each school, whether or not the total number of dengue infections observed were clustered within classrooms. This was done, by comparing the actual distributions of dengue infections among classrooms in each school to an expected Poisson distribution.

### Mapping and geospatial clustering analysis

For clustering analysis, basic mapping of the study area within Plaeng Yao District was performed using Google Earth 7.1 satellite views. After obtaining individual consent, home addresses of the participating students were acquired from school records. Households and schools were geo-referenced using Google Earth with the help and knowledge of local individuals. Geo-referenced households and schools were then mapped using ArcGIS 10.3 (Environmental Systems Research Institute, Inc. Redland, CA, USA). Spatial clustering analysis was done using 50, 100, and 200-metre buffers around households. It evaluated spatial clustering of dengue infections in households using z-scores, assuming that they were randomly distributed among all households. A clustering analysis was performed using a Poisson model with SatScan^TM^ software v9.4.2 (National Cancer Institute, Division of Cancer Control and Population Sciences, Statistical Research and Applications Branch).

## Results

### Dengue infections

Table 1 presents the characteristics of the 10 study schools and their immediate environment. A total of 1,811 students were enrolled in the study, corresponding to an overall participation rate of 78%, ranging from 44% at school S07 (rural) to 94% at school S04 (semi-rural). Twenty-eight students transferred out of the school during the study period. Additionally, 128 students did not provide a second blood sample. Therefore, a total of 1,655 students were included in the analysis of this study. The students resided in 88 classrooms during school hours over the 10 schools. A total of 57 confirmed dengue infections were detected in 7 of the 10 schools (Fig 1). School S03 (semi-rural) had the highest incidence rate of 99 per 1,000 (15 infections among 152 participating students with paired blood samples) during the study. The overall incidence rate for the 10 schools was 34 per 1,000 (57 infections among 1,655 participating students with paired blood samples). Confidence intervals around dengue infections at the school level (Table 1) showed significant differences between some schools when intervals did not overlap. It indicates that school S03 (semi-rural) had a significantly higher infection rate than schools S04 (semi-rural), S06 (rural), S08 (semi-urban) and S10 (semi-urban). It was clearly not the case that dengue infections were randomly distributed over schools, even in this relatively small geographic area.

**Table 1:** School characteristics and summary of dengue infections by school in Plaeng Yao District, Chachoengsao Province.

School Code	Total number of students	Number of participating students (%)	Number with paired blood samples (%)	Number of incident dengue infections	Dengue infection rate /1,000 (95% CI)	Grades taught	Environmental setting of study schools
S01	214	170(79%)	167(98%)	8	48(23 to 88)	Preschool and grades 1–6	Semi-urban with a nearby public park and a lake
S02	44	28(64%)	26(93%)	0	0(0 to 91)	Preschool and grades 1–6	Semi-rural with farm land and farm reservoirs
S03	240	170(71%)	152(89%)	15	99(59 to 154)	Preschool and grades 1–6	Semi-rural with nearby houses and a temple
S04	345	325(94%)	262(81%)	5	19(7 to 41)	Preschool and grades 1–6	Semi-rural with a nearby temple and plantation
S05	48	45(94%)	15(33%)	0	0(0 to 152)	Preschool and grades 1–6	Rural with nearby temple and plantation
S06	194	112(58%)	110(98%)	0	0(0 to 23)	Preschool and grades 1–6	Rural with nearby temple and plantation
S07	169	75(44%)	68(91%)	6	88(38 to 173)	Preschool and grades 1–6	Rural with nearby temple and plantation
S08	416	358(86%)	346(97%)	11	32(17 to 54)	Preschool and grades 1–9	Semi-urban with nearby temple, market and rice paddies
S09	274	234(85%)	224(96%)	9	40(20 to 72)	Preschool and grades 1–9	Semi-urban, next to a village, shop houses and nearby rice paddies
S10	370	294(79%)	285(97%)	3	11(3 to 28)	Preschool and grades 1–6	Semi-urban, in village center with nearby public park and man-made lake

The actual distribution of dengue infections among classrooms in each school was compared with expected Poisson distribution (Table 2). Displayed in Table 2, of the seven schools with dengue infections, three, i.e., schools S03 (semi-rural), S04 (semi-rural) and S08 (semi-urban) had distributions of infections among classrooms that were significantly clustered when compared with the expected Poisson distribution (p<0.001). Overall in the 10 schools, 23 out of the 57 dengue infections (40.0%) occurred in 6 of the 88 classrooms (6.8%).

**Table 2:** Clustering of dengue infections in classrooms within the study schools. Results in bold indicate statistical significance in clustering of dengue infections in classrooms.

School	Number of classrooms with participants	Number of dengue infections in school	Number of classrooms listed according to the number of dengue infections (di)							Comparison of observed vs. Poisson expected
			0 di	1 di	2 di	3 di	4 di	5 di	6 di	
S01	8	8	4	1	2	1	0	0	0	not clustered
S02	5	0	5	0	0	0	0	0	0	-
**S03**	9	15	2	3	3	0	0	0	1	**χ**^**2**^ **= 20.1, p = 0.001**
**S04**	9	5	7	1	0	0	1	0	0	**χ**^**2**^ **= 49.6, p < 0.001**
S05	6	0	6	0	0	0	0	0	0	-
S06	6	0	6	0	0	0	0	0	0	-
S07	6	6	3	1	1	1	0	0	0	not clustered
**S08**	18	11	10	7	0	0	1	0	0	**χ**^**2**^ **= 18.1, p = 0.003**
S09	9	9	3	4	1	1	0	0	0	not clustered
S10	12	3	10	1	1	0	0	0	0	not clustered

### Spatial clustering analyses

For assessing clustering of dengue infections by residential location, it was possible to locate the residences of 39 out of a total of 57 students with dengue infections. Those students who could not be located included five who had moved out of Plaeng Yao District; two who had incomplete addresses in their school records; and 11 whose residences could not be successfully located. A total of 1,035 households of participants without dengue infection were located. Using residential location, we observed that no two infections occurred in the same household. Four infected students resided in households located fewer than 50 metres from each other, and 12 in households within 100 metres of each other. Clustering among residential locations of all participants was then compared to that of participants with dengue infections (<0.0001, z-score = -43.7 vs. <0.001, z-score = -4.58). Results revealed a greater clustering of the households of study participants in general (higher z-score) than those with the dengue infections themselves (lower z-score). Houses in a village are inherently and spatially clustered because of the nature of human settlements and, in Plaeng Yao communities, participants attending the same schools generally resided in the same villages. The z-score results did not, however, suggest that dengue infections were clustered at household level. A further clustering analysis, using the Poisson model in SatScan^TM^ and based on the reference distribution of participants’ households, detected five clusters of dengue infections. Only one cluster was significant (p = 0.006), covering roughly half of the study area. This weak result reflected a lack of substantial clustering among dengue infections when adjusting for the spatial distribution of non-dengue infections. Combined, these three approaches suggest that there is no strong clustering of dengue infections within residential areas.

### Entomological parameters

**Fig 2:**
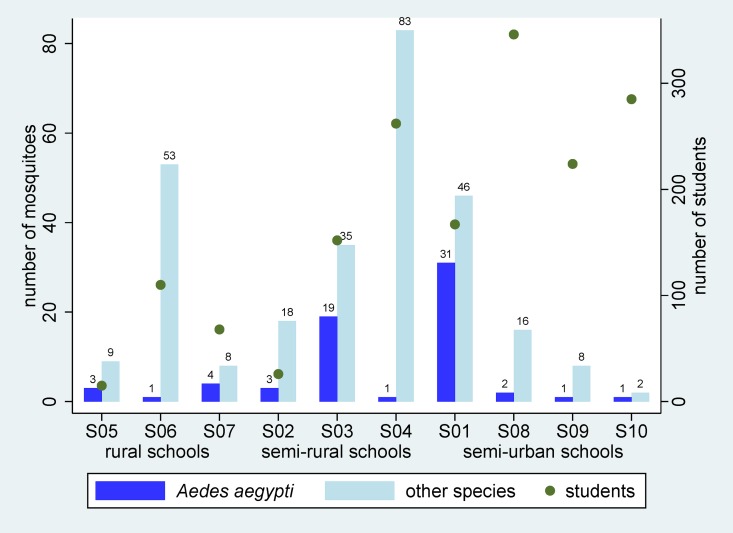
Numbers of mosquitoes caught in schools during school terms using portable vacuum aspirators. Schools are classified by rural, semi-rural or semi-urban status and ranked by the number of students.

**Fig 3:**
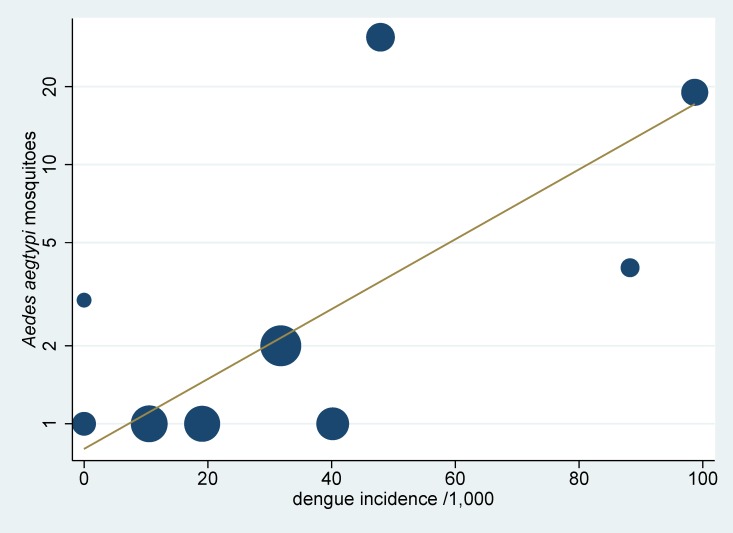
Numbers of mosquitoes caught vs. dengue infections in each school. Each circle represents a school; the size of the circle is proportional to the number of participating students in a school. The regression line is weighted by the number of participating students in each school.

In-classroom mosquito surveys were conducted in June 2012. A total of 66 *Ae*. *aegypti* were identified among 278 mosquitoes (amounting to 24%), collected from 50 classrooms. The highest number of 83 mosquitoes per catch day collected in a school by a portable vacuum aspirator came from school S04 (semi-rural). However, the highest number of *Ae*. *aegypti* collected was 31 from school S01 (semi-urban) (Fig 2). There was a strong and significant correlation between dengue infection and the number of mosquitoes caught by a portable vacuum aspirator in classrooms, as shown in Fig 3 (R^2^ = 0.50, p<0.001), allowing for the difference in the number of participants per school using a weighted regression.

### Breeding site follow-up survey

**Fig 4:**
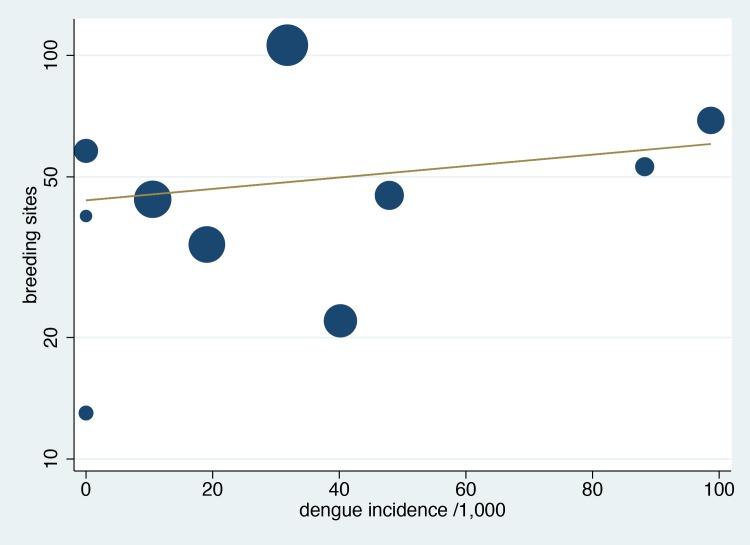
Numbers of active breeding sites of *Aedes* vectors vs. dengue infections in each school. Each circle represents a school, and the size of the circle is proportional to the number of participating students. The regression line is weighted by the number of participating students in each school.

Based on the surveys of breeding sites of *Aedes* vectors after the study, 6 out of the 10 schools were characterized as being located in rural or semi-rural areas surrounded by rice paddies and plantations with nearby villages while four schools were located in a semi-urban setting with household residences and commercial shops located in close proximity but not close enough to be classified as urban (Table 1). A total of 2,399 *Aedes* breeding containers filled with water were identified during the rainy season in all 10 schools during the follow-up breeding site inspection. The school with the highest number (368) of breeding containers with water was school S06 (rural) and that with the lowest number (122) was school S04 (semi-rural). Across all schools, 484 breeding sites (20.2%) were positive for mosquito larvae or pupae. The schools with the highest (106) and lowest (13) numbers of active breeding sites were schools S08 (semi-urban) and S05 (rural), respectively. In a regression of log-transformed number of active breeding sites against dengue infections, weighted for the number of participating students in each school, there was an overall correlation as shown in Fig 4 (R^2^ = 0.03, p <0.001), which was statistically significant but without a very strong association.

**Fig 5:**
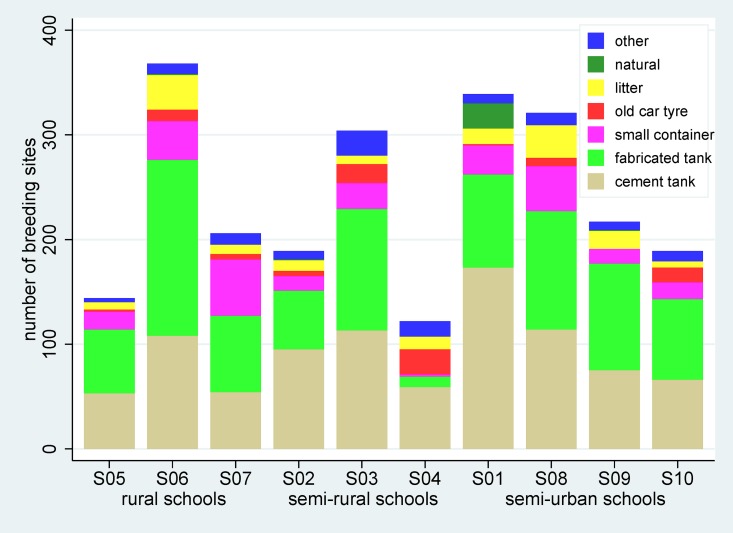
Numbers and types of breeding sites of *Aedes* mosquito vectors in each school.

**Fig 6:**
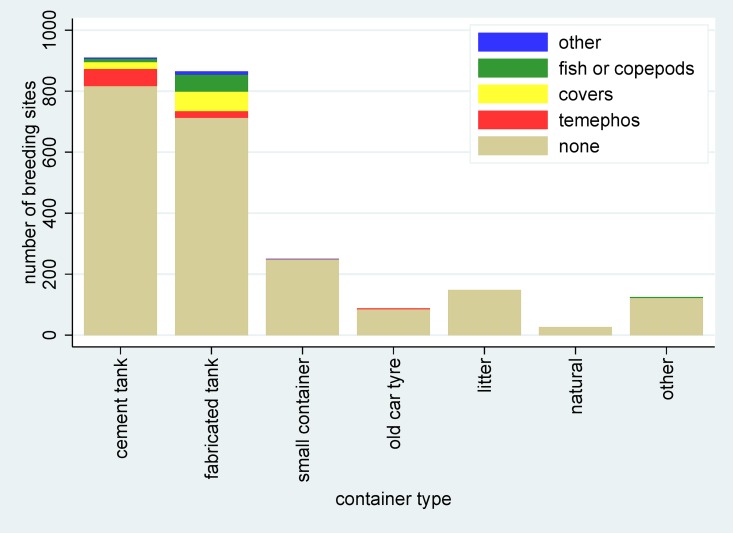
Numbers of breeding sites of *Aedes* mosquito vectors by container type and the extent of vector control measures.

The numbers and types of breeding sites of *Aedes* vectors by school are shown in Fig 5, with substantial differences in overall numbers and types of breeding sites across schools being observed. Among the active breeding sites, the most common types were cement and fabricated water tanks (used for storage of ground water and drinking water respectively) (56.4% combined) and car tyres (8.5%). Vector control measures were only carried out only in 10.7% of all breeding sites, with the use of temephos (32.3%), container covers (30.4%), and larvivorous fish (23.3%) being the most common measures taken, as shown by the container type in Fig 6. Overall, 96% of all vector control efforts were focused at the major breeding sites, primarily cement and fabricated water tanks.

## Discussion

Based on the clustering of dengue infections among school children within school and classroom, the significant correlations between dengue infections and the number of *Aedes* mosquitoes caught in the classrooms together with the active breeding sites in the environment of the schools, our study suggests that schools may be a potential dengue transmission site among school children. Despite the large cluster identified by SatScan as significant, it could not be used as evidence to conclude that dengue infections were actually clustered within the study area by residential location due to its large size covering half of the study area.

The strength of our study is that we identified all incident dengue infections (asymptomatic and symptomatic) through paired blood samples. The large variation of school sizes and participation rates were controlled for, as far as possible, by considering dengue infection rates among participating students. Nonetheless, Fig 3 and Fig 4 show large variability that could explain only in part by the parameters studied. Furthermore, we used a combination of methods that included assessment of mosquito densities, clustering analyse in classrooms, as well as geospatial analyses, in order to identify clustering in residential areas versus schools; all enhanced by surveys to identify *Aedes* breeding sites around schools. Nevertheless, we did not take into consideration dengue infections of people living in the same households or in close proximity to infected children. A further study would be needed to evaluate and clearly compare the importance of transmission site between households and schools.

Our finding that schools are possible sites for dengue transmission is biologically plausible given that *Ae*. *aegypti* bite mainly during the day, with peaks of biting reported between 10.00–11.00 and around 15.00 [12]. Schools in Thailand start around 08.00 and end at 16.00. Because *Ae*. *aegypti* usually have a limited flight range (<100m) [27], a high concentration of this mosquito species would suggest that dengue transmission is likely to occur within the proximity where *Aedes* mosquitoes are caught. Recent studies from Colombia and Mexico also highlighted schools as potential transmission sites using entomological data as their main evidence [20,21]. Olano et al. (2015) performed entomological surveys (both adult and immature forms) in rural schools in Colombia and found the greatest numbers of *Ae*. *aegypti* females within classrooms in comparison to the overall school indoor space. Furthermore, during the wet season, a high infestation with *Ae*. *aegypti* females was observed, with more than half of those caught from schools were in classrooms [21]. Another study by García-Rejón et al. (2011) performed an adult mosquito survey in 24 schools in Mexico and also found the greatest numbers of *Ae*. *aegypti* females in classrooms [20]. Furthermore, García-Rejón et al. stated that, out of a total of 415 *Ae*. *aegypti* females caught, 118 were blood-fed and 16.1% of blood-fed mosquitoes tested positive for DENV infection [20], confirming our study results. Additionally, a large proportion of dengue infections in children are asymptomatic or mild [28–30]. Asymptomatic or mildly symptomatic children who continue to go to school contribute to dengue transmission, as shown in a study in Thailand [29]. However, because the numbers of dengue infection at the classroom level were too small in our study, we had to assume as a null hypothesis that, within each school, classrooms were potentially homogeneous units in terms of how dengue infections in school might be distributed. It was not possible to take classroom location within a school premise into consideration, therefore the reasons for substantial clustering of infections by classroom in some schools remain unexplained.

Through a follow-up survey, we identified the main types of breeding sites for larvae and pupae to be water tanks at 74% of total breeding sites. Among water tanks found, 51% were made of cement and 49% from other types of fabricated materials such as plastic. These tanks were used to store water and required special attention from schools so as to be covered appropriately. The relatively consistent findings between both the number of *Aedes* mosquitoes caught and the breeding sites in relation to dengue infections are encouraging; it could be that the breeding site correlation would have been stronger if the breeding sites containing only *Aedes* vectors were specifically identified. The breeding site data were obtained in the season following the seroconversion data, which might also have diluted the observed effects. In this follow-up survey, we also did not examine the quantitative productivity of the breeding sites.

Only 23 out of 910 cement tanks (2.5%) and 4 out of 865 (7.4%) of other types of water tanks were covered at the time of this inspection, and 89.3% of all breeding sites were not subjected to any vector control method. Our findings of such high numbers of active breeding sites around schools have implications. Schools will need to improve their vector control efforts by targeting these active breeding sites. Among the 11% breeding sites with counter measures, temephos was the most preferred control method used in our study schools. However, temephos resistance among mosquito larvae and water removal from storage containers without replenishing temephos can potentially reduce the effectiveness of this strategy [31,32]. Prevention methods with high visibility and minimal maintenance requirements are highly recommended for schools. Temephos though effective, require regular replenishment for water storage with high water turnover rate. However, temephos is not visible to human-eyes, making it difficult to determine whether or not temephos is already applied at the right concentration in water storages. In our study area, temephos was applied in a sachet which made it possible to be evaluated by our research team. Container covers on the other hand, is highly visible. Similarly, larvivorous fish are also naturally found in Thailand, making it accessible and manageable. Therefore, a wider use of additional methods such as larvivorous fish and container covers should be considered and encouraged to facilitate the control of mosquito breeding sites.

## Conclusion

In summary, our study findings suggest that dengue infections were clustered at the school and classroom levels, implying a possible key role of the school environment in dengue transmission. Study schools were characterized by large numbers and different types of *Aedes* breeding sites. Both *Aedes* mosquito densities and the numbers of breeding sites within schools were significantly correlated with high dengue infections among students. Nonetheless, some dengue infections may also have been acquired outside schools given that infectious mosquito bites may occur outside school hours. Therefore, dengue control efforts should continue to target households, not only to protect children outside of school hours, but also to protect younger children and adults. However, this study underpins the urgency of improving existing vector control strategies at schools, such as indoor residual spraying in classrooms, reduction of breeding sites in school premises, and use of larvicidal methods. However, these approaches have so far failed to successfully control dengue infections in school children. We need to develop novel and complementary vector control measures covering all potential dengue transmission sites, such as impregnated school uniforms and clothing [33,34], impregnated curtains [35], and impregnation of walls [36], or insecticidal methods targeting furniture in schools and households, as well as other innovative non-insecticidal approaches. Further research that aims to compare the significance of households and schools as dengue transmission sites using active surveillance might be strongly beneficial for effective design and budget allocation of future dengue prevention programs.

## Supporting Information

S1 Appendix Data set of serological survey: Data set of serological survey in school children participating in the study (seroconversion status = 1 indicates a positive seroconversion). (XLSX)

S2 Appendix Data set of regression model. Data for regression model with variables: Schools, number of eligible children, actual number of participants, number of blood paired tests, number of positive blood tests, rates and number of *Aedes* mosquitoes captured by aspirator in June 2012.
(XLSX)

S3 Appendix Data set of breeding site survey: Data set of breeding site survey with variables: date, school, container category (1 = cement tank, 2 = fabricated water tank, 3 = small water container, 4 = old car tyre, 5 = litter, 6 = natural container, 9 = others), water level (1 = full container, 2 = 3/4 of the container, 3 = 1/2 of the container, 4 = 1/4 of the container, 5 = less than 1/4 of the container)), mosquito control (0 = none, 1 = temephos, 2 = Bti, 3 = cover, 4 = net cover, 5 = copepods, 6 = fish, 9 = other), and binary (1 = positive) variables for biological control (copepods and fish), container covers and presence of *Aedes* larvae or pupae. (XLSX)

## References

[1] Bhatt S, Gething PW, Brady OJ, Messina JP, Farlow AW, Moyes CL, et al The global distribution and burden of dengue. Nature. 2013;496: 504–507. 10.1038/nature12060 23563266 PMC3651993

[2] Rigau-Pérez JG, Clark GG, Gubler DJ, Reiter P, Sanders EJ, Vance Vorndam A. Dengue and dengue haemorrhagic fever. The Lancet. 1998;352: 971–977. 10.1016/s0140-6736(97)12483-7 9752834

[3] Elling R, Henneke P, Hatz C, Hufnagel M. Dengue Fever in Children: Where Are We Now? Pediatr Infect Dis J. 2013;32: 1020–1022. 10.1097/INF.0b013e31829fd0e9 24008741

[4] Gubler DJ. Dengue, urbanization and globalization: The unholy trinity of the 21st century. Trop Med Health. 2011;39: S3–S11. 10.2149/tmh.2011-S05 PMC3317603 22500131

[5] World Health Organizationon. The Dengue Strategic Plan for the Asia Pacific Region 2008–2015 [Internet]. World Health Organization—South East Asian Region—Western Pacific Region; Available: http://www.wpro.who.int/mvp/Dengue_Strategic_Plan.pdf

[6] Data: Urban Population (% of total) [Internet]. United Nations, World Urbanization Prospects; 2015 Available: http://data.worldbank.org/indicator/SP.URB.TOTL.IN.ZS

[7] WHO. Dengue and severe dengue fact sheet [Internet]. Available: http://www.who.int/mediacentre/factsheets/fs117/en/#

[8] Wichmann O, Hongsiriwon S, Bowonwatanuwong C, Chotivanich K, Sukthana Y, Pukrittayakamee S. Risk factors and clinical features associated with severe dengue infection in adults and children during the 2001 epidemic in Chonburi, Thailand. Trop Med Int Health. 2004;9: 1022–1029. 15361117 10.1111/j.1365-3156.2004.01295.x

[9] Centers for Disease Control and Prevention. Dengue and Dengue Hemorrhagic Fever. 2009.

[10] Gubler DJ. The global emergence/resurgence of arboviral diseases as public health problems. Arch Med Res. 2002;33: 330–342. 12234522 10.1016/s0188-4409(02)00378-8

[11] Moncayo AC, Fernandez Z, Ortiz D, Diallo M, Sall A, Hartman S, et al Dengue emergence and adaptation to peridomestic mosquitoes. Emerg Infect Dis. 2004;10: 1790–1796. 15504265 10.3201/eid1010.030846 PMC3323252

[12] Yasuno M, Tonn RJ. A study of biting habits of *Aedes aegypti* in Bangkok, Thailand. Bull World Health Organ. 1970;43: 319–325. 5312528 PMC2427649

[13] McClelland GAH. Observations on the Mosquito, *Aëdes* (Stegomyia) *aegypti* (L.), in East Africa. I.—The biting cycle in an outdoor population at Entebbe, Uganda. Bull Entomol Res. 1959;50: 227–235.

[14] Gibbons RV, Vaughn DW. Dengue: an escalating problem. BMJ. 2002;324: 1563 12089096 10.1136/bmj.324.7353.1563 PMC1123504

[15] Thai KT, Binh TQ, Giao PT, Phuong HL, Hung LQ, Nam NV, et al Seroprevalence of dengue antibodies, annual incidence and risk factors among children in southern Vietnam. Trop Med Int Health. 2005;10: 379–386. 15807802 10.1111/j.1365-3156.2005.01388.x

[16] Patz JA, Martens WJ, Focks DA, Jetten TH. Dengue fever epidemic potential as projected by general circulation models of global climate change. Environ Health Perspect. 1998;106: 147 9452414 10.1289/ehp.98106147 PMC1533051

[17] Bhumiratana A, Intarapuk A, Chujun S, Kaewwaen W, Sorosjinda-Nunthawarasilp P, Koyadun S. Thailand momentum on policy and practice in local legislation on dengue vector control. Interdiscip Perspect Infect Dis. 2014;2014: 1–11. 10.1155/2014/217237 PMC3995102 24799896

[18] Kittayapong P, Yoksan S, Chansang U, Chansang C, Bhumiratana A. Suppression of dengue transmission by application of integrated vector control strategies at sero-positive GIS-based foci. Am J Trop Med Hyg. 2008;78: 70–76. 18187787

[19] Mammen MP Jr, Pimgate C, Koenraadt CJ, Rothman AL, Aldstadt J, Nisalak A, et al Spatial and temporal clustering of dengue virus transmission in Thai villages. PLoS Med. 2008;5: e205 10.1371/journal.pmed.0050205 18986209 PMC2577695

[20] Garcia-Rejon JE, Lorono-Pino MA, Farfan-Ale JA, Flores-Flores LF, Lopez-Uribe MP, Najera-Vazquez M d. R, et al Mosquito infestation and dengue virus infection in *Aedes aegypti* females in schools in Merida, Mexico. Am J Trop Med Hyg. 2011;84: 489–496. 10.4269/ajtmh.2011.10-0654 21363990 PMC3042828

[21] Olano VA, Matiz MI, Lenhart A, Cabezas L, Vargas SL, Jaramillo JF, et al Schools as potential risk sites for vector-borne disease transmission: Mosquito vectors in rural schools in two municipalities in Colombia. J Am Mosq Control Assoc. 2015;31: 212–222. 10.2987/moco-31-03-212-222.1 26375902

[22] Restrepo BN, Piedrahita LD, Agudelo IY, Parra-Henao G, Osorio JE. Frequency and clinical features of dengue infection in a school children cohort from Medellin, Colombia. J Trop Med. 2012;2012: 1–9. 10.1155/2012/120496 PMC3530854 23304167

[23] Sujariyakul A, Prateepko S, Chongsuvivatwong V, Thammapalo S. Transmission of dengue haemorrhagic fever: At home or school? Dengue Bull. 2005;29: 32.

[24] Wilder-Smith A, Renhorn K-E, Tissera H, Bakar SA, Alphey L, Kittayapong P, et al DengueTools: innovative tools and strategies for the surveillance and control of dengue. Glob Health Action. 2012;5 10.3402/gha.v5i0.17273 PMC3312611 22451836

[25] The Bureau of Registration Administration. Chachoengsao Population Record 2013 [Internet]. [cited 9 Jul 2015]. Available: http://stat.dopa.go.th/stat/statnew/statTDD/views/showDistrictData.php?statType=1&year=56&rcode=24

[26] Johnson AJ, Martin DA, Karabatsos N, Roehrig JT. Detection of anti-arboviral immunoglobulin G by using a monoclonal antibody-based capture enzyme-linked immunosorbent assay. J Clin Microbiol. 2000;38: 1827–1831. 10790108 10.1128/jcm.38.5.1827-1831.2000 PMC86600

[27] Harrington LC, Scott TW, Lerdthusnee K, Coleman RC, Costero A, Clark GG, et al Dispersal of the dengue vector *Aedes aegypti* within and between rural communities. Am J Trop Med Hyg. 2005;72: 209–220. 15741559

[28] Yoon I-K, Srikiatkhachorn A, Hermann L, Buddhari D, Scott TW, Jarman RG, et al Characteristics of mild dengue virus infection in Thai children. Am J Trop Med Hyg. 2013;89: 1081–1087. 10.4269/ajtmh.13-0424 24127167 PMC3854884

[29] Yoon I-K, Rothman AL, Tannitisupawong D, Srikiatkhachorn A, Jarman RG, Aldstadt J, et al Underrecognized mildly symptomatic viremic dengue virus infections in rural Thai schools and villages. J Infect Dis. 2012;206: 389–398. 10.1093/infdis/jis357 22615312 PMC3490697

[30] Peeling RW, Artsob H, Pelegrino JL, Buchy P, Cardosa MJ, Devi S, et al Evaluation of diagnostic tests: Dengue. Nat Rev Microbiol. 2010;8: S30–S37. 21548185 10.1038/nrmicro2459

[31] Braga IA, Mello CB, Montella IR, Lima JBP, Júnior ADJM, Medeiros PFV, et al Effectiveness of methoprene, an insect growth regulator, against temephos-resistant *Aedes aegypti* populations from different Brazilian localities, under laboratory conditions. J Med Entomol. 2005;42: 830–837. 16363168 10.1093/jmedent/42.5.830

[32] Garelli FM, Espinosa MO, Weinberg D, Trinelli MA, Gürtler RE. Water use practices limit the effectiveness of a temephos-based *Aedes aegypti* larval control program in Northern Argentina. Kittayapong P, editor. PLoS Negl Trop Dis. 2011;5: e991 10.1371/journal.pntd.0000991 21445334 PMC3062537

[33] Wilder-Smith A, Lover A, Kittayapong P, Burnham G. Hypothesis: Impregnated school uniforms reduce the incidence of dengue infections in school children. Med Hypotheses. 2011;76: 861–862. 10.1016/j.mehy.2011.02.037 21398046

[34] Paula AR, Carolino AT, Silva CP, Pereira CR, Samuels RI. Testing fungus impregnated cloths for the control of adult *Aedes aegypti* under natural conditions. Parasit Vectors. 2013;6: 256 10.1186/1756-3305-6-256 24010874 PMC3848359

[35] Kroeger 2006 Curtain and cover insec treated.pdf.

[36] Lee HL, Rohani A, Khadri MS, Nazni WA, Rozilawati H, Nurulhusna AH, et al Dengue vector control in Malaysia-challenges and recent advances. Int Med J Malays. 2015;14 Available: http://iiumedic.net/imjm/v1/download/Volume%2014%20No%201/2%20Invited%20Articles/IMJM%20Vol14No1%20Page%2011-16%20Dengue%20Vector%20Control%20in%20Malaysia-%20Challenges%20and%20Recent%20Advances.pdf

